# Persistence of HIV-1 Transmitted Drug Resistance Mutations

**DOI:** 10.1093/infdis/jit345

**Published:** 2013-07-31

**Authors:** Hannah Castro, Deenan Pillay, Patricia Cane, David Asboe, Valentina Cambiano, Andrew Phillips, David T. Dunn, for the UK Collaborative Group on HIV Drug Resistance, Celia Aitken, David Asboe, Daniel Webster, Patricia Cane, Hannah Castro, David Chadwick, Duncan Churchill, Duncan Clark, Simon Collins, Valerie Delpech, Anna Maria Geretti, David Goldberg, Antony Hale, Stéphane Hué, Steve Kaye, Paul Kellam, Linda Lazarus, Andrew Leigh-Brown, Nicola Mackie, Chloe Orkin, Philip Rice, Deenan Pillay, Erasmus Smit, Kate Templeton, Peter Tilston, William Tong, Ian Williams, Hongyi Zhang, Mark Zuckerman, Jane Greatorex, Adrian Wildfire, Siobhan O'Shea, Jane Mullen, Tamyo Mbisa, Alison Cox, Richard Tandy, Tony Hale, Tracy Fawcett, Mark Hopkins, Lynn Ashton, Ana Garcia-Diaz, Jill Shepherd, Matthias L Schmid, Brendan Payne, David Chadwick, Phillip Hay, Phillip Rice, Mary Paynter, Duncan Clark, David Bibby, Steve Kaye, Stuart Kirk, Alasdair MacLean, Celia Aitken, Rory Gunson

**Affiliations:** 1 Medical Research Council Clinical Trials Unit, London, United Kingdom; 2 Division of Infection and Immunity, University College London, London, United Kingdom; 3 Virus Reference Department, Public Health England, London, United Kingdom; 4 Directorate of HIV Medicine and Sexual Health, Chelsea and Westminster Hospital, London, United Kingdom; 5 Research Department of Infection and Population Health, University College London, London, United Kingdom

**Keywords:** persistence, transmitted, HIV-1, resistance, mutations

## Abstract

There are few data on the persistence of individual human immunodeficiency virus type 1 (HIV-1) transmitted drug resistance (TDR) mutations in the absence of selective drug pressure. We studied 313 patients in whom TDR mutations were detected at their first resistance test and who had a subsequent test performed while ART-naive. The rate at which mutations became undetectable was estimated using exponential regression accounting for interval censoring. Most thymidine analogue mutations (TAMs) and T215 revertants (but not T215F/Y) were found to be highly stable, with NNRTI and PI mutations being relatively less persistent. Our estimates are important for informing HIV transmission models.

In Europe around 10% of antiretroviral-naive patients are infected with drug-resistant human immunodeficiency virus type 1 (HIV-1), that is, transmitted drug resistance (TDR) [1]. Because HIV infection is thought to be characterized by a single or narrow spectrum of viruses from the donor, wild-type viral variants are unlikely to coexist with drug-resistant variants, unlike the selection of resistance during treatment. Therefore, the observed rate at which TDR mutations become undetectable (“lost”) is likely to be multifactorial, depending on the number of back mutations required, the relative fitness of mutant and back-mutated viruses, the rate of viral turnover, the presence of compensatory mutations, and the sensitivity of the sequencing assay for detecting low level variants [2–5].

Several studies have reported data on the loss and persistence of TDR mutations; however, the number of patients included in these studies have been small [3, 6]. One larger study (75 patients) quantified the rate of loss of TDR mutations for groups of mutations and found that non-nucleoside reverse transcriptase inhibitor (NNRTI) and protease inhibitor (PI) mutations were lost at a similar slow rate, with a statistically nonsignificant trend toward a higher rate of loss of thymidine analogue mutations (TAMs) and T215 revertants [2]. However, no study has systematically examined and compared the persistence of individual TDR mutations

## METHODS

### Study Population and Definitions

ART-naive patients (both acute/early infection and unknown duration of infection), aged 16 years or older, with TDR mutation(s) detected at their first resistance test (performed between 04/1997 and 09/2009) and who had subsequent resistance test(s) while ART-naive, were identified from the UK HIV Drug Resistance Database [7]. Population sequenced (which detects viral variants above a frequency of 15%–25%) genotypic resistance tests of the *pol* gene were analyzed. The genetic similarity of the sequences from the initial and subsequent resistance tests were compared to exclude super-infection and to check that the samples derived from the same patient. TDR was defined as the presence of ≥1 mutations from the surveillance drug resistance mutations list [8]. Viral subtype was assigned using the REGA algorithm. Demographic and clinical information was acquired by linkage to the UK Collaborative HIV Cohort Study and the UK Register of HIV Seroconverters [7].

### Data Analysis

All analyses were carried out in Stata version 12.0 (StataCorp, College Station, Texas). The rate at which mutations became undetectable (“lost”) was examined using survival models accounting for interval-censored censoring, that is, the exact time the mutation is lost is known only to occur between the last resistance test that detected the mutation and the first test without the mutation (intcens command in Stata). Although a Weibull model indicated a decreasing hazard (results not shown), the parameters from this model lack direct interpretation without knowledge of individuals' dates of infection [2]. We therefore present estimates from the exponential (constant hazard) model; although the data contradict the constant hazard assumption, the estimates can be interpreted as the average rate of loss of mutations following their identification in ART-naive patients during chronic infection.

Mutations with individual frequencies ≥10 (and T215F) were analyzed individually, and those with lower frequencies were grouped by drug class, with the exception of T215 revertants, which were grouped together. An additional analysis examined the effect of patient-level factors on the rate of loss of mutations (accounting for individual mutations), including CD4 cell count and viral load at the first resistance test, viral subtype, first test within 18 months of infection, the number of mutations detected at first test, and whether the mutation was “pure” or part of a mixture. All analyses accounted for multiple mutations at the first resistance test by allowing for within-individual correlation. Finally, we conducted sensitivity analyses removing patients with M184V, those with non-B subtype, and CD4 <200 cells/mm^3^ at the first resistance test, as these factors increase the likelihood that a patient had prior unrecorded ART exposure.

## RESULTS

A total of 313 patients were included in the analysis. Subjects were mainly, but not exclusively, homo/bisexual men infected with a subtype B virus (Table 1). For only a few patients (47; 15%) was the first resistance test known to have been conducted within 18 months of infection. 59% of patients had a single mutation detected at their first test; 27% and 6% had mutations conferring resistance to two and three ART classes, respectively. Of the total 717 TDR mutations detected at the first resistance test, 147 (21%) were present as a mixture (92 with wild-type amino acid alone, 37 with a non-TDR mutation alone, 18 with both). Most patients (279; 89%) had only one resistance test following the initial test which detected TDR mutations and before starting ART; the median (interquartile range [IQR]) interval between tests was 40 (10–96) weeks.

**Table 1.** Description of Study Population and Initial Resistance Test

	N (%) or Median (IQR)
No. of patients	313
Gender	
Male	220 (70)
Female	22 (7)
Unknown	71 (23)
Exposure source	
Homo/bisexual	187 (60)
Heterosexual	24 (8)
Other (including 1 injecting drug user)	13 (4)
Unknown	89 (28)
CD4 at first test (cells/mm^3^)^a^	427 (268, 545)
Viral load at first test (log_10_ copies/mL)^b^	4.6 (4.0, 5.1)
Subtype	
B	248 (79)
Non-B	42 (13)
Not classified	23 (7)
First test within 18 mo of infection^c^	
No or unknown	266 (85)
Yes	47 (15)
No. of mutations in first test	
1	185 (59)
2	59 (19)
3	23 (7)
≥4	46 (15)
No. of patients with	
≥1 NRTI mutation	204 (65)
≥1 NNRTI mutation	120 (38)
≥1 PI mutation	74 (24)
No. of patients with resistance to	
1 class	212 (68)
2 classes	83 (27)
3 classes	18 (6)

Abbreviations: HIV, human immunodeficiency virus; IQR, interquartile range.

^a^ Within 90 days before/after resistance test, N = 217.

^b^ Within 90 days before/after resistance test, N = 238.

^c^ First resistance test within 18 months of HIV-negative test in patients with ≤18 months between HIV-negative and HIV-positive tests.

### Rate of Loss of Individual TDR Mutations

The overall rate of loss of mutations was 18 (95% confidence interval [CI], 14–23) per 100 person-years of follow-up (PYFU), although the rate varied considerably for individual mutations (Table 2). Within drug class, NRTI mutations showed the most variation in persistence (heterogeneity *P* < .001). As expected, M184V was lost rapidly at a rate of 71 (95% CI, 34–149) per 100 PYFU. M41L was commonly observed and highly persistent (rate of loss 8 (95% CI, 4–15) per 100 PYFU), and a similar low rate of loss was seen for other TAMs (D67N, L210W, and K219Q/N); however, K70R appeared to be lost more quickly. There was also a rapid transition of T215F and T215Y to one of the T215 revertants, but the revertants themselves were highly stable with a rate of loss of 5 (95% CI, 3–11) mutations per 100 PYFU. Consequently, there was a large number of T215 revertants at the initial resistance test.

**Table 2.** Rate of Loss of TDR Mutations

Mutation	No. of mutations at first resistance test	No. (%) of mutations which became undetectable	Rate of loss (95% CI) (per 100 PYFU)	Median time to loss (years) (95% CI)
All	717	171 (24)	18 (14–23)	3.9 (3.0–5.0)
Any NRTI	401	90 (22)	15 (11–21)	4.6 (3.3–6.4)
M41L	77	11 (14)	8 (4–15)	8.6 (4.6–16.0)
D67N	27	4 (15)	12 (4–33)	6.0 (2.1–16.9)
K70R	14	7 (50)	38 (17–83)	1.8 (.8–4.0)
M184V	34	16 (47)	71 (34–149)	1.0 (.5–2.0)
L210W	25	6 (24)	14 (6–33)	4.8 (2.1–11.2)
T215Y	25	13 (52)	41 (20–84)	1.7 (.8–3.4)
T215F	9	4 (44)	58 (15–224)	1.2 (.3–4.6)
T215 revertants	106	9 (8)	5 (3–11)	13.0 (6.6–25.7)
K219Q	25	2 (8)	4 (1–19)	15.8 (3.6–70.0)
K219N	12	2 (17)	15 (3–72)	4.6 (1.0–22.4)
All other NRTI^a^	47	16 (34)	22 (12–38)	3.2 (1.8–5.6)
Any NNRTI	154	37 (24)	25 (17–38)	2.7 (1.8–4.1)
K103N	73	12 (16)	18 (10–34)	3.7 (2.0–6.8)
Y181C	20	10 (50)	54 (26–113)	1.3 (.6–2.7)
G190A	17	4 (24)	19 (6–56)	3.6 (1.2–15.5)
All other NNRTI^b^	44	11 (25)	27 (13–54)	2.6 (1.3–5.3)
Any PI	162	44 (27)	21 (14–31)	3.3 (2.2–4.9)
M46L	16	5 (31)	22 (8–59)	3.1 (1.2–8.4)
I54V	16	5 (31)	21 (8–50)	3.3 (1.4–7.8)
V82A	16	3 (19)	13 (5–39)	5.1 (1.8–14.8)
I84V	10	3 (30)	20 (5–76)	3.4 (.9–12.9)
L90M	32	5 (16)	12 (5–31)	5.8 (2.2–15.3)
All other PI^c^	72	23 (32)	28 (17–46)	2.5 (1.5–4.1)

Abbreviations: CI, confidence interval; NRTI, nucleoside reverse transcriptase inhibitors; NNRTI, non-NRTI; PYFU, person-years of follow-up; PI, protease inhibitors; TDR, transmitted drug resistance.

^a^ K65R(3), D67E(1), D67G(6), T69D(7), 69 insertion(T)(1), K70E(1), L74I(3), L74 V(3), V75A(2), V75M(2), V75 T(2), Y115F(1), Q151M(1), M184I(2), K219E(6), K219R(6).

^b^ L100I(3), K101E(9), K101P(3), K103S(4), V106A(2), V106M(4), Y181 V(1), Y188L(8), G190E(2), P225H(5), M230L(3).

^c^ L24I(2), D30N(2), V32I(3), M46I(8), I47A(1), I47 V(1), G48 V(4), I50 V(2), F53L(6), I54A(2), I54L(3), I54 T(2), G73S(6), G73 T(2), V82F(2), V82L(9), V82S(1), V82 T(4), N83D(2), I85 V(6), N88D(3), N88S(1).

There was no statistically significant difference in the rate of loss of NNRTI mutations (heterogeneity *P* = .1); K103N was the most common NNRTI mutation, with a rate of loss of 18 (95% CI, 10–34) mutations per 100 PYFU. NNRTI mutations appeared to be lost more quickly than most TAMs (M41L, D67N, L210W, and K219Q/N) and the 215 revertants (*P* < .001 for both comparisons). L90M was the most common PI mutation, with a rate of loss of 12 (95% CI, 5–31) mutations per 100 PYFU. However, there was little variation in the rate of loss across PI mutations (heterogeneity *P* = .6), with a rate of loss similar to that of most of the NNRTI mutations. Sensitivity analyses removing patients with M184V (n = 34), those with non-B subtype (n = 42), or patients with CD4 < 200 cells/mm^3^ at the first resistance test (n = 29) resulted in slightly lower absolute rates of TDR mutation loss but did not materially affect comparisons within and between drug classes.

### Predictors of the Rate of Loss of TDR Mutations

In multivariate analysis, there was no clear effect on the rate of loss of TDR mutations of CD4 cell count (*P* = .5) or viral load (*P* = .2) at the first resistance test, recent infection (*P* = .3), or the number of mutations detected at the first test (*P* = 1.0). A statistically significant higher rate of loss was seen with non-B subtype infection than subtype B infection (adjusted hazard ratio = 2.8, 95% CI, 1.2–6.3, *P* = .01), and also, as expected, if the TDR mutation at the first resistance test was present as a mixture (adjusted hazard ratio = 6.8, 95% CI, 4.2–11.2, *P* < .001).

## DISCUSSION

By including patients with unknown duration of infection as well as those identified during acute/early infection, this is the largest study to date to provide quantitative estimates of the persistence of individual TDR mutations. Wide variability in persistence was observed for NRTI mutations in particular, highlighting the need to be careful when grouping mutations for the purposes of analysis. In a recent study of patients with acute/early infection all TAMs were combined for analysis, but we found marked variation within this group of mutations, with T215F/Y and K70R being lost more rapidly than other TAMs [2]. However, T215 revertants were highly persistent, consistent with the fitness advantages associated with this evolutionary pathway [9]. M184V was lost rapidly, although at a lower rate than reported by Jain et al [2], possibly reflecting a selection bias in our analysis. Lesser heterogeneity was observed for NNRTI and PI mutations, and mutations from these classes were lost more rapidly than the T215 revertants and the more stable TAMs, such as M41L. This is in contrast to previous smaller studies, which have generally observed NNRTI mutations to be relatively stable [3, 10], and also to the study by Jain et al, which reported a trend toward a higher rate of loss of TAMs and T215 revertants compared to NNRTI mutations [2].

Transmission of TDR occurs both from ART-experienced patients with acquired resistance as well as onward transmission from ART-naive individuals. Our finding that certain mutations are highly stable and not replaced by wild-type virus, along with high levels of viral suppression among patients receiving ART, suggests that TDR may increasingly stem from the ART-naive population. HIV transmission models are critical for predicting future levels and patterns of TDR, and TDR persistence among ART-naive patients is a key component of these [11, 12]. Because of the lack of epidemiological data, Wagner et al [12] used estimates of fitness costs from viral competition experiments [13] to calibrate their models. They reported that at least 2 mutations (K70R and Y181C) could form self-sustaining transmission chains. However, there is a discord between our empirical estimates of persistence of individual TDR mutations with in vitro fitness cost estimates. For example, certain TAMs and PI mutations were more stable than would be expected, given their highly impaired replicative impairment [13, 14]. The determinants of persistence of specific viral species in vivo will not only include complex genetic interactions (eg, compensatory mutations [4, 5]) but also other aspects of host-pathogen biology, such as immune responses.

Our finding that some TDR mutations may persist for several years supports the continued use of baseline genotypic resistance testing in chronically infected patients. It is also important to note that the marked variability in the persistence of individual TDR mutations indicates that the detection of ≥1 mutations may signal that viruses harboring other undetected mutations could have been archived in latent cells and thus affect response to subsequent ART.

We found no effect of CD4 cell count or viral load on the persistence of TDR mutations, in agreement with Bezemer et al [6], although the rate of loss was higher in patients with non-B subtype infection than subtype B. Although there is no obvious virological explanation for this finding, one possibility is differential ART misclassification by patient characteristics linked to viral subtype. The rate of loss of mutations was similar if the mutation detected at the first test was present in isolation or accompanied by other mutations; further analyses are planned to look at the role of compensatory mutations [4, 5].

To maximize the information available we included patients with unknown duration of infection, as well as patients identified during acute/early infection. This introduces a selection effect because some potentially eligible patients will have lost TDR mutations before their first resistance test. Only including patients identified during acute infection would minimize this effect, although, even then, some highly unfit mutations such as M184V could still be missed. Another limitation of the analysis is that population sequencing was used to detect mutations rather than more sensitive methods capable of detecting minor variants, and therefore we may be overestimating the rate of loss [15].

In summary, this is the first study to our knowledge to provide estimates of the persistence of individual TDR mutations. The disconnect with in vitro estimates of resistance-associated viral fitness costs underlines the key role of epidemiological data in calibrating HIV transmission models, which are critical for predicting the future course of the TDR epidemic.
